# Human enterovirus 71 epidemics: what's next?

**DOI:** 10.3402/ehtj.v6i0.19780

**Published:** 2013-09-10

**Authors:** Cyril C. Y. Yip, Susanna K. P. Lau, Patrick C. Y. Woo, Kwok-Yung Yuen

**Affiliations:** 1 Department of Microbiology, The University of Hong Kong, Hong Kong, China; 2 State Key Laboratory of Emerging Infectious Diseases, Department of Microbiology, The University of Hong Kong, Hong Kong, China; 3 Research Centre of Infection and Immunology, The University of Hong Kong, Hong Kong, China; 4 Carol Yu Center for Infection, The University of Hong Kong, Hong Kong, China

**Keywords:** human enterovirus 71, hand, foot and mouth disease, evolution, genotype, mutation, recombination

## Abstract

Human enterovirus 71 (EV71) epidemics have affected various countries in the past 40 years. EV71 commonly causes hand, foot and mouth disease (HFMD) in children, but can result in neurological and cardiorespiratory complications in severe cases. Genotypic changes of EV71 have been observed in different places over time, with the emergence of novel genotypes or subgenotypes giving rise to serious outbreaks. Since the late 1990s, intra- and inter-typic recombination events in EV71 have been increasingly reported in the Asia-Pacific region. In particular, ‘double-recombinant’ EV71 strains belonging to a novel genotype D have been predominant in mainland China and Hong Kong over the last decade, though co-circulating with a minority of other EV71 subgenotypes and coxsackie A viruses. Continuous surveillance and genome studies are important to detect potential novel mutants or recombinants in the near future. Rapid and sensitive molecular detection of EV71 is of paramount importance in anticipating and combating EV71 outbreaks.

Human enterovirus 71 (EV71) is a small, non-enveloped, icosahedral virus that belongs to the human EV species A in the genus *Enterovirus* within the family *Picornaviridae*. EV71 and coxsackievirus A16 (CVA16) are common etiological agents of hand, foot and mouth disease (HFMD) in children, but the former can cause severe complications, such as aseptic meningitis, acute flaccid paralysis (AFP), meningoencephalitis and cerebellitis, with mortality rate ranging from 10 to 25.7% (1, 2). Another neurotropic EV, poliovirus, is nearly completely eradicated as a result of global immunization efforts (3). Thus, in the absence of effective vaccines and antivirals against EV71, EV71 may become an important pathogen, replacing poliovirus, with increasing health threat to humans. Since the late 1990s, EV71 has seriously affected the Asia-Pacific region (4–10). In recent years, there have been an increasing number of reports of HFMD outbreaks with fatal cases due to EV71 in China (11–18). In 2012, EV71 was found to be associated with ‘mystery disease’ that killed most patients shortly after admission to hospital in Cambodia (19). EV71 is well known to cause outbreaks, which often occur in a cyclical pattern, every 2–3 years, in various countries (20).

The single-stranded positive-sense RNA genome of EV71 is around 7.4-kb long, which is flanked by 5′ and 3′ untranslated regions (UTRs). The polyprotein consists of P1, P2, and P3 regions, which encodes structural proteins, VP4, VP2, VP3, VP1, and non-structural proteins, 2A, 2B, 2C and 3A, 3B, 3C, 3D, respectively. Based on molecular typing using VP4 and VP1 gene sequences (21), EV71 is classified into three genotypes, A, B (subgenotypes B1–B5), and C (subgenotypes C1–C5) (21, 22). A separate subgenotype B0 has recently been identified in a retrospective analysis of EV71 strains in the Netherlands from 1963 to 1967 (23). Two studies on complete genome analysis of EV71 strains of subgenotype C4 suggested that this subgenotype should be classified as a novel genotype D (24, 25).

Mutation and recombination are well-known phenomena in EV evolution. The infidelity of EV 3D polymerase leads to their mutation rates of around one per genome per replication (26). Mutations in various regions such as 5′UTR, VP1, VP2, 2A, 2C, and 3D of EV71 have shown to be associated with alterations of virulence in animal models and humans (27–33). Recombination occurs in enteroviruses as a result of template switching during negative-strand synthesis, which is thought to be mediated by a ‘copy-choice’ mechanism (34, 35). Inter-typic and intra-typic recombination events were frequently detected in EV71 strains circulating in the Asia-Pacific region (24, 36–38). In recent years, recurring HFMD outbreaks caused by EV71 of subgenotype C4 (‘double-recombinant’ belonging to a novel genotype D) have been reported in Hong Kong and different provinces in China (11–16, 39). In this review, we provide an update on the epidemiology and genetic evolution of EV71.

## Methods

Keywords including ‘EV71’, ‘EV71’, ‘EV’, ‘epidemiology’, ‘evolution’, ‘genotype’, ‘mutation’, and ‘recombination’ were used for Medline search. The search results were then manually screened for literature on the epidemiology and genetic evolution of EV71.

### Epidemiology of EV71

Many studies have reported the detection of EV71 in clinical specimens of patients from various countries in Asia, Australia, Europe, and America (Table 1) (40–47). During 1969–1972, the prototype EV71 strain BrCr and related strains were first identified from patients with neurological disease in California (40). In the early 1970s, EV71 occurred in patients mainly with meningitis in USA, Sweden, and Australia (41–43). A large outbreak of poliomyelitis-like disease occurred in Bulgaria in 1975, during which 21% of around 700 cases showed paralytic forms and 27 were fatal as a result of EV71 infections (44, 48, 49). Three years later, EV71 was the major causative agent of meningitis and encephalitis during the severe epidemic of acute central nervous system (CNS) diseases in Hungary (45). During the 1970s, Japan experienced two outbreaks of HFMD by EV71, in which a significant proportion of patients with HFMD accompanied CNS disorders and some of them died (46, 50, 51). Based on the above findings, EV71 isolates in America, Australia, and Europe were strongly associated with severe CNS complications, while those in Japan showed both dermatotropic and neurotropic features. Mortality rates due to EV71 outbreaks in the 1980s were low when compared to those occurred in Bulgaria and Hungary in the 1970s (52).

**Table 1:** Summary of major EV71 outbreaks in different geographical regions

Time period	Country/region	No. of cases with EV71	No. of deaths due to EV71	Clinical findings (no. of cases)	Genotype(s)	References
1969–1972	California	20	1	Meningitis (10), encephalitis (7), meningoencephalitis (1), myocarditis (1)	A	40, 79
1972	New York	11	0	Meningitis (9), encephalitis (1), HFMD (1)		41
1972–1973	Australia	49	0	Aseptic meningitis (39), rash alone (5), acute RTI (4), infective polyneuritis (1)		43
1973	Sweden	195	0	Mainly aseptic meningitis, some with HFMD		42
1975	Bulgaria	65 (by virus isolation); 282 (by serology)	27	Aseptic meningitis (30), bulbar forms with fatal outcome (27), poliomyelitis syndrome (8)		48
1977	New York	12	0	CNS disease (7), HFMD (4), acute RTI (1), gastroenteritis (1)		71
1978	Hungary	323	47 (not clear if all due to EV71)	Meningitis (161), encephalitis (145), poliomyelitis (13), HFMD (4)		45
1973, 1978	Japan	71	Some died (details not given)	Mainly HFMD, some with CNS disorders		46
1986	Australia	114	0	Rash (61), RTI (35), meningoencephalitis (34)		64
1988–1990	Brazil	39	0	Acute neurological disease (24)		149
1997	Malaysia	2,628 HFMD cases	29	Cardiorespiratory failure (29)		53
1997–1998	Singapore	39	0	Mainly HFMD, some with aseptic meningitis, AFP, myocarditis, coxsackie-like disease and neonatal pyrexia		150
1998	Taiwan	469	34	HFMD or herpangina, 78 with severe complications (mainly encephalitis)		54, 61–63
1997–2000	Peninsular Malaysia	43 strains	0	HFMD (33), encephalitis (4), myocarditis (2), meningitis (1), HFMD with meningitis (1), oral ulceration (1), paralysis (1)	B3, B4, C1, C2	6
1998–2000	Taiwan	340 (53 isolates for phylogenetic study)	7 (from phylogenetic study)	HFMD (20), HFMD with CNS involvement (12), meningoencephalitis (5), encephalitis (4), meningitis (2), CNS symptoms (1), HFMD with acute pharyngitis and asthmatic bronchitis (1), HFMD with acute gastritis (1), herpangina (1)	Mainly C in 1998, B in 1999 and 2000	8
1999	Perth	14	0	Meningitis (5), acute cerebellar ataxia (2), acute transverse myelitis (2), Guillain–Barré syndrome (2), benign intracranial hypertension (1), febrile convulsion (1), opso-myoclonus syndrome (1)		68
2000	Korea	12	0	HFMD (8), HFMD and encephalitis (2), HFMD and bronchiolitis (1), AFP (1)	C	106
2000	Singapore	81	4	HFMD (76), non-HFMD [aseptic meningitis, herpangina, Guillain–Barré syndrome] (5)	B4	7
1998–2003	Japan	110 strains	0	HFMD (95), URTI (4), influenza-like illness (3), herpangina (3), exanthema (3), meningitis (2)	B4, B5, C2, C4	65
2000–2003	Malaysia	277	4	Mild HFMD (187), HFMD with CNS involvement (52), severe non-CNS HFMD (34), aseptic meningitis (4)	B4, B5, C1	66
2005	Vietnam	173	3	HFMD (173), of which 51 complicated by acute neurological disease	C1, C4, mainly C5	10
2006	Brunei	34	2	HFMD or herpangina	B4, mainly B5	95
1986, 2007	The Netherlands	>40 in 1986, 58 in 2007	0	Mainly fever, meningitis or encephalitis, gastrointestinal symptoms	B2 in 1986, mainly C2 in 2007	23
2006–2007	France	28	1	Mainly fever, some with HFMD, meningitis, acute respiratory distress syndrome, gastroenteritis	C1 and C2 in 2006, C2 in 2007	60
2007	Germany	13	0	Meningitis (9), HFMD (1)	C2	58
2007	Denmark	17	0	Meningitis (5), HFMD (4), unspecified viral infection (3), gastroenteritis (2), viral enteritis (1), HFMD with meningitis (1), meningitis with enteritis (1)	B5, C1, C2	59
2008	Hong Kong	98	1	HFMD (89), herpangina (2), fever, URTI, rash and pneumonia (7) (11.2% of the cases with complications including meningitis or encephalitis, pneumonia, AFP and shock)	B5, C2, mainly C4	39
2008	China—Fuyang	59	6	HFMD: mild (17), severe (36), fatal (6)	C4	11
2008	China —Guangdong	551	21	Complete data available for 185 cases: 95% rash; encephalitis (7), paralysis (1), neurogenic pulmonary edema (5), pneumonia (4)	C4	12
2008–2009	Thailand	23	1	Mainly HFMD, brainstem encephalitis (2)	B5, C1, C2, mainly C4	9
2009	China—Beijing, Shandong, Guangdong	134	0	HFMD	C4	13
2009–2010	China—Shanghai	378	Some died (details not given)	HFMD, some with CNS involvement	C4	14
2010	China—Nanchang	63	0	HFMD	C4	15
2008–2011	China—Ningbo	1,503	10	HFMD: mild (1,349), severe (144), fatal (10)	C4	16

AFP=acute flaccid paralysis; CNS=central nervous system; HFMD=hand, foot and mouth disease; URTI=upper respiratory tract infections.

Since the late 1990s, recurrent EV71 epidemics of various scales have occurred in the Asia-Pacific region, including Australia, China, Malaysia, Singapore, Taiwan, Thailand, and Vietnam (4–10). A huge number of deaths associated with HFMD outbreaks occurred in Malaysia, Taiwan, and China. During the outbreak with 2,628 HFMD cases in Sarawak Malaysia, 29 children died due to rapidly progressive cardiorespiratory failure caused by EV71 in 1997 (53). In Taiwan, 78 of 405 children with severe complications died in the large HFMD outbreak in 1998 (54), followed by another outbreak in 2000 with 41 deaths among 80,677 HFMD cases (8). During these two outbreaks, EV71 accounted for a major proportion of deaths in children in Taiwan. Since May 2008, HFMD has been a notifiable disease in the national surveillance system in China (17). From 2008 to 2011, recurring HFMD outbreaks have occurred in various provinces in China and the number of HFMD cases increased from 488,955 with 126 deaths to 1,619,706 with 509 deaths (11–18, 55). During this period, coxsackieviruses A2, A4, A5, A6, A10, A12, A16, and EV71 were co-circulating in the outbreaks (13, 14, 56), of which EV71 was responsible for most fatal cases (16).

The number of EV71-associated HFMD cases was relatively low in Europe compared to that in the Asia-Pacific region. In a prospective study from Norway (57), EV71 was detected in stool specimens from asymptomatic children, and the absence of disease may be due to host factors (immune system, genetic effect, nutritional and hygiene status) and/or viral factors. Since HFMD is not a disease under surveillance in Europe and healthy individuals are usually not the subjects under surveillance, the prevalence of EV71 may be underestimated (23, 58–60).

EV71 epidemics usually occur in summer months. In studies with clinical specimens collected throughout the year, seasonal patterns of EV71 infection have been demonstrated. Several studies showed that EV71 could be detected throughout the year, but its predominance was found in different months in various regions (9, 10, 57). A higher incidence was observed in summer months in Norway (57), but in the fall in Vietnam and Thailand (9, 10). Some countries have shown two peak activities of EV71 infections during their study periods. In 1998, there were two peaks of EV71 infections (one in June and the other in October) in the large outbreak in Taiwan (61–63). In southern Vietnam, a smaller peak (March–May) and a higher peak (September–December) of EV71 infections were found in 2005, and these months are interim periods between the dry and wet seasons (10). In Hong Kong, a higher peak (May–June) and a smaller peak (October–December) have been noted in 2008 (39). In the Netherlands, a higher peak (June–July) and a smaller peak (September–October) of cases were observed during 1963–2008 (23). Some studies have also reported variation of peak season between different years. In Australia, peak activity of EV71 shifted from summer in 1973 to winter in 1986 (43, 59, 64). In Japan, EV71 was detected in summer months in 1998–1999, but was only detected in the fall during 2001–2002 (65). In Malaysia, EV71 predominance shifted from summer in 2000 to spring in 2003 (66). The observed seasonal changes may be due to climatic factors that favor viral survival, variations in host immune response to infection and host behaviors that increase contact between individuals.

### Clinical impact of EV71 infection

EV71 infection usually causes HFMD or herpangina (54, 67), but it can result in more severe illness, which is characterized by high-grade fever (body temperature above 39°C), vomiting, and cardiopulmonary or neurological complications (54). In the large-scale EV71 epidemic in Taiwan in 1998, 78 patients died with severe illnesses, including AFP, aseptic meningitis, encephalitis, pulmonary edema or hemorrhage, and myocarditis, among whom around 80% had pulmonary edema or hemorrhage (54). During an HFMD outbreak in Australia in 1999, there was a study showing that 14 children with EV71 infection had neurological complications including meningitis, acute cerebellar ataxia, acute transverse myelitis, Guillain–Barré syndrome, benign intracranial hypertension, febrile convulsion, and opso-myoclonus syndrome (68). In a follow-up study of 142 children after EV71 infection with CNS involvement, neurological disease and cardiorespiratory failure were likely associated with neurologic sequelae (limb weakness and atrophy), delayed neurodevelopment, and reduced cognitive function (69). In 2012, more than 50 children, who presented with fever, respiratory illness, and neurological complications, died within a short period of time after admission to hospital in Cambodia, where EV71 was eventually identified as a possible cause of the outbreak (70).

### Detection of EV71

Traditional methods of EV71 detection are virus isolation and serological tests (40, 41, 45, 46, 48, 64, 71, 72). For cell culture, human rhabdomyosarcoma (RD) and monkey kidney cell lines (e.g., Vero) are commonly used to isolate EV71 (1), but this method is rather time-consuming (take days to weeks) and has poor sensitivity (73). For serology, cases were reported as positive when paired sera from patients showed fourfold increase in neutralizing antibody titers against EV71 (40, 44–46, 48). However, acute and convalescent sera are usually taken at least 2 weeks apart (74), rendering serological tests on paired sera, together with virus isolation, unsuitable for managing EV71 in outbreak situations, during which early detection of EV71 is required to allow prompt implementation of preventive and control measures. Since the late 1990s, rapid and sensitive molecular diagnostic tests such as reverse-transcriptase polymerase chain reaction (RT-PCR) have been increasingly applied for EV71 detection. In epidemiological studies during the HFMD outbreaks, 5′UTR and VP1 were the most widely used targets for EV71 detection (Table 2). 5′UTR was used because this region showed high sensitivity for the detection of EV71 (61, 73, 75). However, 5′UTR is a hot spot of recombination in enteroviruses (76, 77), making this region inappropriate for genotyping (21). In contrast, VP1 gene is most commonly used for phylogenetic analysis as it shows a high degree of genetic diversity and no homologous recombination has been reported to take place within the VP1 gene in EV71 (78). To better determine the prevalence of EV71 in clinical specimens, 5′UTR should be used for detection and VP1 for genotype and subgenotype classification.

**Table 2:** Summary of studies on the phylogeny of EV71 strains

Gene or region	Number of nucleotides sequenced	Number of new strains for phylogenetic analysis (country/collection date)	References
5′UTR	440	13 (Malaysia/1997)	AbuBakar et al. (75)
VP1	891	113 (USA, Australia, Colombia, the Republic of China, Canada, Malaysia/1970–1998)	Brown et al. (79)
VP4/VP2	420	29 (Malaysia, Japan/1997; Taiwan/1998; Bulgaria, Hungary, Japan, Taiwan, USA/1973–1980)	Shimizu et al. (151)
5′UTR	681	36 (Taiwan/1998)	Wang et al. (61)
VP1	529–633	16 (Taiwan/1998)	Shih et al. (97)
VP1	341	20 (Japan, Malaysia, Singapore/1997–1998)	Singh et al. (150)
VP4	207	3 (Taiwan/1986); 20 (Taiwan/1998)	Chu et al. (98)
VP1	891	24 (Malaysia/1997–1998, 2000); 19 (Singapore/1998, 2000–2001); 23 (Australia/1999–2000)	McMinn et al. (4)
5′UTR and VP1	648 and 841	48 (Taiwan/1998–2000) for 5′UTR; 33 (Taiwan/1998–2000) for VP1	Wang et al. (8)
5′UTR, VP4 and VP1	646, 207 and 855	1 (India/2001)	Deshpande et al. (114)
VP4 and VP1	207 and 891	55 (USA, Malaysia, Singapore, Australia, Korea/1972–2002) for VP4; 12 (Korea/2000; Malaysia/2002) for VP1	Cardosa et al. (21)
VP1	891	11 (Korea/2000)	Jee et al. (106)
VP1	891	43 (Malaysia/1997–2000)	Herrero et al. (6)
VP1	891	5 (China/1997, 2000); 1 (Thailand/2002)	Shimizu et al. (84)
VP4 and VP1	207 and 891	17 for VP4 and 19 for VP1 (China-Shenzhen/2001–2004)	Li et al. (5)
VP1	891	1 (Brazil/1999)	Castro et al. (113)
VP1	891	45 (Japan-Yamagata/1998–2003)	Mizuta et al. (65)
VP4	207	121 (Japan-Fukushima/1983–2003)	Hosoya et al. (83)
VP4	207	41 (Taiwan/1986, 1999–2005)	Lin et al. (85)
VP1	891	48 (Australia-Sydney/1983–2001)	Sanders et al. (86)
VP1	414	85 (Taiwan/1998–2005)	Kung et al. (101)
VP4 and VP1	207 and 840–891	7 for VP4 and 14 for VP1 (Malaysia-Sarawak/2000, 2003)	Ooi et al. (66)
VP1	partial-891	1 with complete genome; others with partial VP1 (data not shown) (Norway/2002–2003)	Witsø et al. (57)
VP1	891	23 (Vietnam/2005)	Tu et al. (10)
VP1 and 3Dpol	891 and 1391	32 (the United Kingdom/1998–2006)	Bible et al. (88)
VP1	403	11 (Taiwan/2006–2007)	Huang et al. (22)
5′UTR and VP1	723 and 891	16 (Austria/2001–2004)	Ortner et al. (89)
VP1	891	199 (The Netherlands/1963–2008)	van der Sanden et al. (23)
VP1	891	26 (Japan-Toyama/1983, 1989, 1994, 1997, 2000, 2003, 2006)	Iwai et al. (87)
VP1	891	31 (Japan-Yamagata/1990–2007)	Mizuta et al. (104)
VP1	372	28 (Germany/1997–2007)	Diedrich et al. (58)
VP1	837–891	34 (Brunei/2006); 7 (Malaysia/2006)	AbuBakar et al. (95)
VP1	891	56 (China-Shandong/2007)	Zhang et al. (110)
VP1	891	3 (France/2007–2008)	Vallet et al. (105)
VP1	255	6 (Hungary/2000, 2004–2005)	Kapusinszky et al. (82)
VP1	436–458	3 (Korea/2003)	Jeong et al. (107)
VP1	403	11 (Taiwan/2008)	Huang et al. (100)
VP1	891	5 (China-Lu'an/2008)	Yu et al. (81)
VP1	891	10 (Singapore/2008)	Wu et al. (93)
VP1	158–159	23 (Thailand/2008–2009)	Chatproedprai et al. (9)
VP1	891	28 (China-Jiangsu/2009)	Mao et al. (112)
VP1	891	58 (France/1994, 1999–2000, 2003–2009)	Schuffenecker et al. (60)
VP1	891	31 (China/2003, 2008–2009)	Tan et al. (17)
VP2	159	29 (Denmark/2005–2008)	Badran SA et al. (59)
VP1	NA	44 (Taiwan/2008)	Lee et al. (99)
VP1	891	17 (China-Guangdong/2008)	Sun et al. (12)
VP1	403–891	6 (Thailand/2008–2009, 2011)	Puenpa et al. (92)
VP1	891	20 (China-Henan/2009)	Zhang et al. (111)
VP1	891	8 (China-Shanghai/2009)	Yan et al. (14)
VP1	304	17 (China/2009)	Yang et al. (13)
VP1	315–706	6 (Greece/2009–2010)	Siafakas et al. (109)
VP1	891	4 (China–Nanchang/2010)	Liu et al. (15)
VP1	NA	78 (China-Ningbo/2008–2011)	Ni et al. (16)

NA: not available.

### Genotypic changes in EV71

**Fig. 1:**
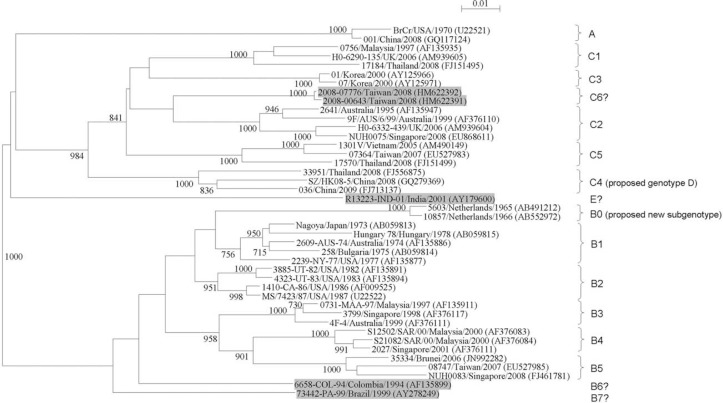
Phylogenetic tree of the VP1 region of EV71 strains detected in various countries, showing different genotypes and subgenotypes of EV71. Eight hundred and fifty-five nucleotide positions in each VP1 region were included in the analysis. The tree was constructed by the neighbor joining method and bootstrap values calculated from 1,000 trees. The scale bar indicates the estimated number of substitutions per 100 nucleotides. EV71 strains of potential novel genotype or subgenotype were highlighted in gray. GenBank accession numbers are indicated in parentheses.

Based on molecular characterization using VP1 gene sequences, EV71 was classified into genotypes A, B, and C (Fig. 1) (79). The prototype strain BrCr isolated in 1970 during the epidemic in California was classified as genotype A (40, 79, 80). There was no report on the circulation of EV71 genotype A strains thereafter until 2008 when re-emergence of this genotype occurred in central China (81). Genotype A might not be the first genotype detected in human population because a novel subgenotype of EV71 circulating in the Netherlands during 1963–1967 was recently identified as subgenotype B0 (23), which existed earlier than genotype A. Since the 1970s, EV71 strains of genotype B (subgenotypes B1–B5) have been circulating globally. Subgenotype B1 was the major type responsible for EV71 epidemics in America (79), Europe (6, 23, 82), Asia (21, 83–85) and Australia (23, 79) in the 1970s, while subgenotype B2 became predominant in the United States (79), the Netherlands (23), Australia (86), and Japan (83) in the 1980s. In the mid-1980s, EV71 strains of subgenotype C1 emerged and have been circulating in different regions afterwards (Table 3) (4, 9, 10, 21, 23, 39, 57–60, 79, 82–92).

**Table 3:** Summary of EV71 subgenotypes in different geographical regions from 1963 to 2011

	Subgenotypes											
	
Year	A	B0 (proposed)	B1	B2	B3	B4	B5	C1	C2	C3	C4 (proposed genotype D)	C5
1963		NL										
1965		NL										
1966		NL										
1967		NL										
1970	**USA**		JPN									
1971			NL									
1972			NL, USA									
1973			JPN, NL, TW	JPN								
1974			AUS, NL	AUS								
1975			**BUL**, NL									
1976			NL, USA									
1977			USA	NL								
1978			**HUN**, NL, USA, **JPN**									
1979			NL, USA									
1980			**TW**, USA									
1981				NL, USA								
1982			JPN	NL, USA								
1983			JPN, USA	AUS, JPN, NL, USA								
1984			**JPN**									
1985			JPN	NL								
1986			**TW**, USA	**NL**, USA				AUS				
1987			USA	USA				AUS, NL, USA				
1988				USA				HK, USA				
1989				JPN				AUS, JPN, USA				
1990				JPN				AUS, JPN, NL, USA				
1991								AUS, CAN, NL, USA				
1992								AUS, NL, USA				
1993				JPN	JPN			JPN, USA				
1994								AUS, FRA, HK, NL, USA				
1995								AUS, NL, USA	AUS			
1996					JPN			AUS, JPN	CHN, UK			
1997				GER	JPN, **MAL**, SIN	**JPN**, **MAL**, SIN		NL, MAL, USA	**JPN**, HK, NL, MAL, UK, USA		JPN	
1998				GER	SIN	TW		MAL, SIN, UK	AUS, JPN, **TW**, UK, USA		CHN, HK, TW	
1999					**AUS**, HK, SIN	MAL, TW		FRA, HK, MAL, UK	**AUS**, JPN, FRA, NL, UK		HK	
2000						AUS, **JPN**, **MAL**, **SIN**, **TW**	SIN	AUS, FRA, GER, HK, HUN, MAL, UK	FRA, JPN, NL	**KOR**	CHN, HK	
2001						AUS, HK, SIN, **TW**		AUT, GER, NL, TUR, UK	FRA, JPN		CHN, HK, TW	
2002						JPN, MAL, SIN, TW		AUT, HK, MAL, NL, NOR, SIN, THA, UK	HK, JPN, NL		CHN, JPN	
2003						JPN, MAL, TW	**JPN**, **MAL**	AUT, FRA, GER, JPN, MAL, NOR	CHN		CHN, HK, **JPN**, KOR	
2004								FRA, NL, UK	NL		AUT, CHN, FRA, GER, JPN, HK, HUN, **TW**	
2005							**MAL**	FRA, IRAN, NL, **MAL**, UK, VNM	CHN,			
DEN,												
NL		CHN, HUN, HK, **TW**, VNM	**VNM**									
2006						BRU	**BRU**, MAL, **SIN**	FRA, UK	CHN, DEN, FRA, GER, UK		CHN, HK, JPN, THA	TW
2007							DEN, TW	DEN, NL	CHN, DEN, FRA, GER, HK, **NL**, THA		**CHN**, HK, JPN	TW
2008	CHN						HK, **SIN**, **TW**	THA	CHN, DEN, FRA, HK, NL, SIN, THA, UK		**CHN**, HK, TW, THA	TW
2009							THA, TW	THA	CHN, FRA, GRE, KOR, THA		**CHN**, KOR, THA	
2010									CHN, GRE		**CHN**, TW	
2011							THA				**CHN**	

AUS=Australia; AUT=Austria; BRU=Brunei; BUL=Bulgaria; CAN=Canada; CHN=China; DEN=Denmark; FRA=France; GER=Germany; GRE=Greece; HK=Hong Kong; HUN=Hungary; JPN=Japan; KOR=Korea; MAL=Malaysia; NL=The NL; NOR=Norway; SIN=Singapore; TW=Taiwan; THA=Thailand; TUR=Turkey; UK=The United Kingdom; USA=The United States of America; VNM=Vietnam.

Boldface denotes predominant subgenotype causing large outbreak.

The emergence of a novel genotype or subgenotype of EV71 can lead to large outbreaks, with well-known examples in the Asia-Pacific region since 1997 (Table 3). In Malaysia, a widespread community HFMD outbreak with fatal cases occurred in 1997, during which the most prevalent subgenotype B3 was co-circulating with other subgenotypes B4, C1, and C2 (6, 21, 23, 53, 79, 85, 91). Subgenotypes B4 and C1 were identified as a cause of an outbreak in 2000 (6, 91), while subgenotype B5 was noted in an outbreak in 2005 (65, 66, 85, 91). In Australia, co-circulation of subgenotypes B3 and C2 contributed to a large outbreak in 1999, with subgenotypic changes to B4 and C1 in 2000 (4, 21, 23, 85, 86). In Singapore, three major outbreaks have been caused by subgenotypes B4 (2000) and B5 (2006 and 2008) (4, 85, 93, 94). Brunei reported its first EV71 outbreak in 2006, of which most EV71 isolates belonged to subgenotype B5 (95). In Taiwan, the predominant subgenotype C2 accounted for a devastating EV71 outbreak in 1998, followed by a shift to subgenotype B4 (1999–2003), C4 (2004–2005), C5 (2006), B5 (2007–2009), and C4 (2010) (8, 21–23, 85, 96–103). Intriguingly, similar pattern of inter-genotypic change (a change from one genotype to another) of EV71 has been observed in Japan in outbreaks since 1997 (65, 83–85, 87, 104).

Inter-genotypic change of EV71 has also been detected in Europe. In the Netherlands, genotype B (subgenotypes B0, B1, and B2) from 1963 to 1986 was changed to genotype C (subgenotypes C1 and C2) since 1987 (23). In Denmark, only subgenotype C2 was identified in 2005–2006, with a shift to predominant subgenotype B5 in 2007 (59). In Germany, a shift from subgenotype B2 (1997–1998) to subgenotype C1 (2000–2003) has been noted, followed by intra-genotypic change (a change from one subgenotype to another of the same genotype) to C4 (2004) and C2 (2006–2007) (58). Intra-genotypic change of EV71 has been found in some other European and Asian countries. Two studies demonstrated that there was a shift from subgenotype C1 (2001–2003 in Austria; 2000 in Hungary) to C4 (2004 in Austria; 2004–2005 in Hungary) (82, 89). During 2000–2009, all EV71 strains belonged to genotype C in France, in which C1 strains predominated from 2000 to 2005, but C2 strains became predominant since 2007 (60, 105). In Korea, subgenotype C3 was identified in an EV71 epidemic in 2000, followed by the detection of subgenotype C4 in 2003 and 2009 (21, 85, 106–108).

Circulation of a single EV71 subgenotype was reported in several places. In Iran, only EV71 of subgenotype C1 was detected from a child with AFP in 2005 (90). In Greece, all EV71 strains detected from children with HFMD, febrile illness, or maculopapular rash belonged to subgenotype C2 in 2009–2010 (109). Coexistence of more than one subgenotype was observed in some other regions (Table 3). During 1998–2006, subgenotype C1 predominated in the United Kingdom, with the existence of subgenotype C2 in 1999 and 2006 (88). The first report of subgenotype C5 in Vietnam showed that this predominant subgenotype was co-circulating with subgenotypes C1 and C4 in 2005 (10). In Thailand, subgenotypes C1 and C4 were identified in 2008, followed by the detection of subgenotypes C1, C2, C4, and B5 in 2009 (9, 84, 92, 110). In China, the major subgenotype C4, which was further divided into C4a (2002–2011) and C4b (1998–2004), co-circulated with subgenotype C2 (5, 12–17, 84, 110–112). Similar subgenotype predominance was noted in Hong Kong (situated on China's south coast), where the main subgenotype of EV71 belonged to C4, with co-detection of a minority of subgenotypes B3, B4, B5, C1, and C2 strains during 1998–2008 (39).

Identification of EV71 genotype or subgenotype has not been standardized yet, although several studies have proposed certain criteria for typing. Distinct clusters with high bootstrap supports in phylogenetic trees constructed by using VP1 gene indicated the presence of a novel genotype or subgenotype of EV71 (4, 79). Furthermore, cutoff values for genotyping and subgenotyping of EV71 using VP1 have been proposed, with a nucleotide sequence divergence of 15–20% between genotypes and of 4–14% between subgenotypes (25, 79, 101). According to these criteria, previously untyped EV71 isolates may belong to a novel genotype or subgenotype. Two studies demonstrated that an EV71 strain 6658-COL-94 from Columbia and a strain 73442-PA-99 from Brazil were closely related to genotype B isolates (79, 113). Based on phylogenetic analysis using VP1 nucleotide sequences of EV71 (Fig. 1), the two strains formed branches separate from other genotype B strains. Moreover, VP1 sequence divergence between the Colombian strain and other genotype B strains was 6.1–12% and between the Brazilian strain and other genotype B strains was 8.2–12%, suggesting that they should belong to novel subgenotypes B6 and B7, respectively. As shown in Fig. 1, EV71 strains 2008-00643 and 2008-07776 from Taiwan (100) formed a cluster distinct from subgenotype C2 isolates. In addition, the VP1 sequence divergence between theses Taiwanese strains and other genotype C strains is 6.7–13.6%. These indicated that the two Taiwanese strains should be regarded as a new subgenotype C6. Phylogenetic analysis of 5′UTR, VP4 and VP1 sequences of an EV71 strain R13223-IND-01 isolated from a case of AFP in India in 2001 showed that it may belong to a novel genotype (114). As shown in Fig. 1, this isolate formed a branch distinct from other EV71 strains. Furthermore, the VP1 sequence divergence between this Indian strain and other strains of EV71 genotypes A, B, and C was 14.9–18.5%. These revealed that the strain R13223-IND-01 should be designated as a new genotype E. Recently, Chan et al. proposed new cutoff values for genotyping based on complete genome sequences of EV71, with a nucleotide divergence of 17–22% between genotypes and of 10–14% between subgenotypes (25). Complete genome sequencing and further sequence analysis are required to better determine the genotype or subgenotype of EV71.

### Relationship between EV71 genotypes and disease severity

Several research groups have examined the relationship between EV71 genotypes and outcomes of EV71 infections. In a study from Australia, EV71 of subgenotype C2 was strongly linked to severe neurological disease in 1999 (4). Another study from Malaysia revealed that children infected with EV71 of subgenotype B4 were less likely to have CNS complications than those infected with other subgenotypes (66). However, a recent study from the Netherlands demonstrated that children with genotype B virus were more likely to have neurological complications than those with genotype C virus (23). In Taiwan, EV71 of genotypes B and C could be isolated from both fatal and mild HFMD cases from 1998 to 2000 (8, 97). Furthermore, VP1 sequences of EV71 isolated from patients with mild HFMD and fatal encephalitis in Malaysia in 1997 were almost identical (79). Hence, more careful and systematic analysis will be required to delineate the correlation between the EV71 genotypes and disease severity.

### Recombination events in EV71

**Fig. 2:**
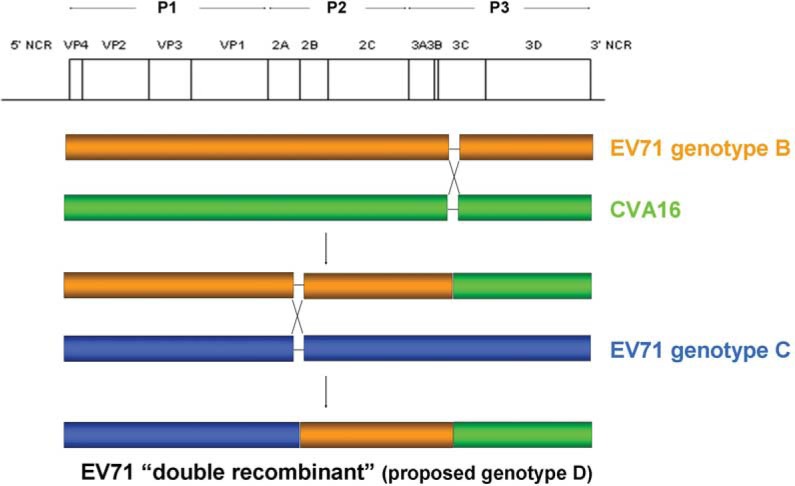
Schematic diagram showing intra- and inter-typic recombination events occurred in EV71 strains SZ/HK08-5 and SZ/HK08-6 of subgenotype C4 (proposed genotype D).

Under selective pressures from hosts and environment, recombination possibly takes place when at least two viruses infect the same cell, resulting in the production of progenies with new genomic combinations that may favor viral survival during evolution (115). As recombination in RNA viruses might enhance their host range (116) and pathogenicity (117), and confer antiviral resistance (118), it is important to assess the impact of recombination in EV71. Recombination is a common phenomenon in human enteroviruses, with preferential recombination sites in non-structural protein coding regions P2 and P3 (Fig. 2), where a high nucleotide sequence identity between two parental strains may favor homologous recombination by a ‘copy-choice’ mechanism (34, 35). Breakpoints are frequently detected at 5′UTR, P2 and P3 by recombination analysis using sequencing of different gene regions or complete genomes (24, 37, 38, 76, 77, 119–123). In a recent study using VP1 and 3D for recombination analysis on 308 EV71 isolates collected from 19 countries over a 40-year period, 11 3D clades were identified, each specific to EV71 and associated with specific subgenotypes but interspersed phylogenetically with clades of CVA16 and other HEV-A serotypes (124). Sporadic recombination events were detected within genotypes but no evidence for inter-typic recombination as described in other studies (24, 36, 37, 125). Since recombination breakpoints can occur at 5′UTR and different gene regions of EV71, amplification of two gene regions (VP1 and 3D) may not be sufficient to reveal the actual recombination events.

Recent studies on EV71 recombination usually involve complete genome sequence analysis. During the HFMD outbreaks in Malaysia in 1997, EV71 subgenotype B3 was predominant, which was also found in other areas in the Asia-Pacific region including Japan, Singapore, Australia, and Hong Kong (4, 21, 23, 36, 39, 83, 85) (Table 3) but disappeared after 1999. Complete genome analysis of two strains SHA63 and SHA66 showed that they were most closely related to EV71 genotype B strains for 5′UTR, P1 and P2 regions, but to CVA16 for the P3 and 3′UTR (36). This was the first to demonstrate inter-typic recombination (between different types of human enteroviruses, e.g., EV71 and CVA16). Since 1998, emergence of EV71 subgenotype C4 has been responsible for the HFMD outbreaks in China. Phylogenetic and bootscan analyses on two complete genomes of EV71 C4 strains SHZH98 and SHZH03 from Shenzhen revealed that they were most closely related to EV71 subgenotype C2 strain for P1 region, but to CVA16 for P3 region (37). In the same study, a CVA16 strain Tainan5079 was closely related to CVA16 strain G-10 for P1 region, but to EV71 strain BrCr of genotype A for P2 and P3 regions. These indicated that inter-typic recombination events have occurred between EV71 subgenotype C2 and CVA16 strain G-10, and between CVA16 strain G-10 and EV71 genotype A, both with a breakpoint at 2A region, a recombination hot spot in enteroviruses (1, 120, 121).

Various recombinant forms of EV71 accounted for outbreaks in Taiwan since 1998. Inter-typic recombination between EV71 and coxsackievirus A8 (CVA8) was detected in EV71 subgenotype C2 isolates that were responsible for the large outbreak associated with severe encephalitis in 1998 (96), which was in line with a previous study (126). During 2000–2001, outbreaks in Taiwan were caused by EV71 subgenotype B4, which was possibly evolved from genotypes B3 and B2 (96). From 2004 to 2005, the predominant EV71 subgenotype C4 emerged and was shown to be a recombinant resulting from intra-typic recombination (between the same type of human EV, e.g., EV71) between genotype C and genotype B (96), consistent with a previous study (38).

In China, a dramatic increase in the number of HFMD cases from 2007 to 2008 suggested EV71 and CVA16 might have undergone recombination (24). Complete genome analysis of two EV71 strains (SZ/HK08-5 and SZ/HK08-6) and two CVA16 strains (SZ/HK08-3 and SZ/HK08-7) from Shenzhen revealed inter-typic recombination between CVA16 strain G-10 and EV71 genotype A at the 2A–2B junction the two CVA16 strains (24), in line with previous results on CVA16 strain Tainan5079 (37). For the two EV71 strains, intra-typic recombination between EV71 genotypes C and B at 2A–2B junction and inter-typic recombination between EV71 genotype B and CVA16 strain G-10 in 3C region were observed (Fig. 2). These ‘double-recombinant’ EV71 strains circulating in China and other EV71 subgenotype C4 strains were proposed to be a novel genotype D (24). The proposal of this new genotype was also supported by another study conducted by Chan et al. (25). In this study, EV71 subgenotype C4 was shown to have a nucleotide sequence divergence of 18.1% (17–20%), which exceeded the average threshold divergence of 14.95% for EV71 subgenotyping when comparing with EV71 subgenotypes C1–C5. Based on the evidence from the two studies (24, 25), EV71 subgenotype C4 should be redesignated as the novel genotype D. Since 2008, there has been an increase in the number of studies on recombination in EV71 during HFMD outbreaks in China, including the ‘double-recombinant’ subgenotype C4 strains probably belonging to the proposed genotype D (11, 125, 127). Although the correlation between natural recombination in EV71 and pathogenicity remains uncertain, an *in vitro* study demonstrated that a chimeric recombinant virus with improved growth and larger plaque phenotypes could be artificially constructed by replacing the structural region of a slow-growth EV71 strain with the region of a rapid-growth EV71 strain (128). Thus, it is possible to generate a highly pathogenic EV71 strain when a less virulent strain can acquire an antigenically distinct capsid region or non-structural regions from a more virulent strain via natural recombination.

Over the past decade, EV71 of predominant subgenotype C4 has been co-circulating with some other subgenotypes in mainland China and Hong Kong (5, 17, 23, 39, 129), which may increase the chance of recombination. In addition, densely populated areas with poor hygiene, sanitation, and healthcare infrastructure may further hasten not only recombination, but also viral mutation. As Hong Kong is a gateway to China with extensive passenger movements and global transport networks, it may be a place for the spread of novel EV71 mutants or recombinants to other cities, posing pandemic threats as in the case of SARS.

### Antiviral strategies against EV71

Despite the occurrence of recurrent EV71 outbreaks with severe complications and fatal cases in the past few decades, effective antivirals against EV71 are still not available (130). Intravenous immunoglobulin (IVIG) has been used in patients with complicated EV71 infections, which may help suppress viral replication and limit organ damage through anti-inflammatory activities (66). *In vitro* and *in vivo* studies demonstrated that ribavirin and type I interferons exhibited protective effects on EV71 (131, 132). Pleconaril has demonstrated antiviral activity against a broad spectrum of EV serotypes *in vitro* and *in vivo* (133), but it cannot inhibit the cytopathic effect induced by EV71 (134). In a study by Shih et al., mutation in VP1 of EV71 was shown to confer resistance to the inhibitory effects of pyridyl imidazolidinone (135). EV71 mutants resistant to inhibitors of 2C protein of EV71, including metrifudil, N(6)-benzyladenosine and NF449, have also been identified (136). In another study, Chen et al. demonstrated that EV71 displayed resistance to an antiviral agent DTriP-22 after an arginine-to-lysine substitution (R163K) in 3D polymerase (137). So far, none of these antivirals possessed efficacy high enough for clinical use.

Due to the high frequency of mutations and recombination in EV71, viral factors may not be suitable targets for drug design. In contrast, targeting cellular factors temporarily dispensable for the host but essential for viral replication may prevent viral escape. RNA interference (RNAi) screening has been increasingly used to search for cellular factors required for viral infections (138–147) and this strategy holds a potential for antiviral development (148). Further investigations to identify host factors important for EV71 replication will help explore the mechanisms of EV71 pathogenesis.

## Concluding remarks

Over the past few decades, EV71 epidemics have occurred in various countries and caused a significant proportion of severe complications and deaths in children, particularly in the Asia-Pacific region. Mutation and recombination are the major evolutionary forces leading to emergence of genetically diverse EV71 variants that have accounted for the recurrent HFMD outbreaks. Despite recent findings of intra-typic and inter-typic recombination, the correlation between recombination and virulence in EV71 remains unclear. Owing to the common occurrence of recombination in EV71, sequencing of more than one region (e.g., VP1 and 3D) would allow the rapid and accurate genotyping of EV71 in clinical settings. To date, the majority of HFMD cases due to EV71 have been noted in China, which is the most populous country in the world. Since Hong Kong is well connected to China with international travel networks, the former may be a hub that facilitates the global dissemination of novel mutants or recombinants of EV71, posing pandemic threats in the near future. Continuous genomic studies on the evolution of EV71 in Hong Kong and other Asia-Pacific regions are important to detect new mutants or recombinants with epidemic potential. Frequent genetic variations in EV71 have hampered the development of drugs targeting to viral proteins and this obstacle could theoretically be overcome by targeting host factors that are inessential for humans but important for virus propagation. Genome-wide RNAi screening technology has successfully been applied for the identification of cellular factors crucial for replication of emerging viruses, such as HIV and influenza viruses. We foresee that this screening strategy will help unravel EV71–host interactions and provide insight into the discovery of novel antivirals to combat future EV71 epidemics.
